# IL-2 and Mycobacterial Lipoarabinomannan as Targets of Immune Responses in Multiple Sclerosis Patients

**DOI:** 10.3390/microorganisms8040500

**Published:** 2020-04-01

**Authors:** Marco Bo, Magdalena Niegowska, Jessica Frau, GianPietro Sechi, Giannina Arru, Eleonora Cocco, Leonardo A. Sechi

**Affiliations:** 1 Department of Biomedical Sciences, Division of Microbiology and Virology, University of Sassari, 07100 Sassari, Italy; 30039536@studenti.uniss.it (M.B.); Magda@uniss.it (M.N.); 2 Multiple Sclerosis Center, Binaghi Hospital, ATS Sardegna, Department of Medical Sciences and Public Health, University of Cagliari, 09126 Cagliari, Italy; jessicafrau@hotmail.it (J.F.); ecocco@unica.it (E.C.); 3 Clinic of Neurology, Department of Clinical, Surgical and Experimental Sciences, University of Sassari, 07100 Sassari, Italy; parentina@yahoo.com (G.A.); gpsechi@uniss.it (G.S.)

**Keywords:** interleukin 2, IL-2, multiple sclerosis, antibodies, autoimmune response, *Mycobacterium avium* subsp. paratuberculosis, HERV-W

## Abstract

Interleukin 2 (IL-2) is considered a key player in exacerbating multiple sclerosis (MS). Therapies targeting its receptor have been developed; however, a resolution of the disease and side effects are still an issue of concern. The involvement of other factors, such as *Mycobacterium avium* subspecies *paratuberculosis* (MAP) and envelope protein derived from human endogenous retrovirus type W (HERV-Wenv), in MS pathogenesis has been recently suggested. Here, we investigated the levels of antibodies (Abs) directed against IL-2 and HERV-Wenv in 108 MS patients, 34 patients affected by neuromyelitis optica spectrum disorder (NMOSD), and 137 healthy controls (HCs). Our results show increased levels of Abs specific to IL-2 and HERV-Wenv-su antigens in MS vs. HCs (*p* < 0.0001 for IL-2, *p* = 0.0004 for HERV-Wenv) and significantly decreased levels in NMOSD vs. MS. The assessment of different 12-month-long therapies on Abs against IL-2, HERV-Wenv, and MAP lipoarabinomannan (LAM) demonstrated the strongest effect on anti-LAM Abs (*p* = 0.018), a slight reduction of anti-IL-2 Abs, and small variations for anti-HERV-Wenv Abs. These results highlight the conclusion that the impact of therapy is more correlated with selected epitopes than with the therapeutic agent. Screening for anti-IL-2 and anti-HERV-Wenv Abs has a potential as additional future practice to distinguish between symptomatically similar MS and NMOSD.

## 1. Introduction

The interplay between genetic and environmental factors leading to the development of multiple sclerosis (MS) is currently a commonly accepted scenario. Even though high-risk genetic variants have been identified, definitive evidence confirming a causative contribution of exogenous agents is still missing. The risk of MS is reported to increase several fold following delayed primary infection by Epstein–Barr virus (EBV), known as infectious mononucleosis [1]. However, no studies have proven the EBV-specific expression in MS, nor has the presence of EBV been demonstrated convincingly in the MS brain. We previously showed that *Mycobacterium avium* subsp. *paratuberculosis* (MAP) may be another infectious agent implicated in MS development as anti-MAP antibodies (Abs) targeting peptides homologous to EBV epitopes are enriched in the blood and cerebrospinal fluid (CSF) of MS patients [2], possibly contributing to the autoimmune process through cross-reactivity that results from erroneous recognition between exogenous and self-antigens. Some families of human endogenous retrovirus (HERV) have recently emerged as possible components involved in the process leading to autoimmunity. It is supposed that certain silenced genes of HERV integrated in multiple copies across the human genome may, under largely unknown circumstances including infections, be transactivated and generate antigens triggering abnormal immune responses [3,4,5,6]. In particular, hyperexpression of the envelope protein belonging to HERV family W (HERV-Wenv) has been associated with autoimmune diseases [5,7].

The unclear MS etiology results in the lack of therapy able to restore the function of neurons damaged during the pathological process. In turn, currently employed treatment is symptomatic and aims at speeding recovery from immune attacks on the central nervous system (CNS), thereby only delaying MS progression that in numerous cases is insufficient to prevent progressive disabling forms of the disease. Moreover, incorrect therapy may be administered to patients whose clinical outcomes are confounded with initially similar disorders, such as neuromyelitis optica spectrum disorder (NMOSD) [8].

In recent years, interleukin 2 (IL-2) has been suggested to play a key role in MS etiopathogenesis by regulating immune cell responses, and its elevated expression in Th17 lymphocytes, along with other factors promoting inflammation, could be related to high inflammatory status [9]. Low doses of IL-2 have been successfully employed as add-on MS therapy [10], while the detection of increased IL-2 levels in MS patients has led to the development of therapeutic approaches targeting IL-2 receptor [11]. However, further observations have demonstrated that side effects such as severe inflammatory brain disorders [12,13] and resistance to antagonistic antibody therapies that target receptors at the cell surface may arise in a relatively short time [14]. Moreover, high expression of IL-2 has been reported in animals after *Mycobacterium tuberculosis* and MAP infection [15,16]. At the same time, little attention has been paid to anti-IL-2 antibodies (Abs) detected in a range of autoimmune diseases [17]. The functional capacity of these anti-IL2 antibodies—at the monoclonal level—to neutralize or otherwise modulate IL-2 function remains to be assessed.

In this study, we evaluated the levels of Abs against two IL-2 peptides and antigens deriving from the surface portion of HERV-Wenv (HERV-Wenv-su) and lipoarabinomannan (LAM) of MAP in Sardinian MS patients with a common high-risk haplotype. Even though a conspicuous body of literature describes the association of both agents with the disease, few studies are focused on the impact that MS therapy has on specific Abs. Our previous results demonstrated that a two-year-long natalizumab treatment is able to effectively reduce the levels of Abs against HERV-W and MAP [18]. Here, we assessed changes in humoral responses involving autoreactive and HERV-W/MAP-specific Abs patterns following a 12-month therapy with fingolimod, teriflunomide, interferon beta (INF-β), or the lack of treatment.

## 2. Materials and Methods

### 2.1. Study Population

In all, 108 MS patients (66 females, 42 males; mean age 40.06 ± 13.90) diagnosed according to the revised McDonald diagnostic criteria [19] were enrolled at the Multiple Sclerosis Centre of the University of Cagliari, Cagliari, Italy, and the Neurology section of the University of Sassari, Sassari, Italy. Of these, 34 patients underwent a relapse in the following 12 months (classified as relapsing-remitting multiple sclerosis (RRMS)), 4 had their MS criteria changed to secondary progressive multiple sclerosis (SPMS), and 70 were at onset (Table 1). Ten MS patients with HLA-DRB1*0301-DQB1*0201 haplotype that confers a high risk of developing the disease [20], (9 females, 1 male; mean age 44.98 ± 11.55) were kept under observation to evaluate effects of MS therapy on serological outcomes. Follow-up samples were available after one year without therapy (*n* = 3) or following administration of natalizumab (*n* = 2), teriflunomide (*n* = 2), fingolimod (*n* = 1), or INF-β (*n* = 2). In addition, 34 NMOSD patients (5 males, 29 females; mean age 51.32) at disease onset and diagnosed based on established criteria [21] and free from immunomodulatory therapy over the last 12 months were enrolled at the Neurology Clinic of the University Hospital of Sassari, Italy. All NMOSD sera were tested for the presence of Abs to aquaporin-4 (AQP4) through the commercial Anti-Aquaporin-4 IIFT screening test (Euroimmun, Luebeck, Germany) [22]. Finally, 137 reference control subjects (90 females, 47 males; mean age 46.30 ± 12.72) were enrolled from among voluntary blood donors attending the Transfusion Unit of the University Hospital of Sassari, Italy.

Demographic and clinical features of study participants are presented in Table 1.

### 2.2. Antigens and Serological Testing

Synthetic peptides IL-2_6–20KK_ (KK-LLSCIALSLALVTNS-KK) and IL-2_56–70_ (LTEMLTFKFYMPKKA) were commercially obtained at >90% purity (LifeTein, South Plainfield, NJ, USA) based on Pérol et al. [17] with modifications and used for detection of relative antibodies in patients’ sera. Responses to retroviral peptides HERV-Wenv-su_93–108_ (NPSCPGGLGVTVCWTY) and HERV-Wenv-su_248–262_ (NSQCIRWVTPPTQIV) corresponding to surface portions of the HERV-W envelope glycoprotein, which previously showed a high immunogenic activity in MS patients, were assessed in parallel. Additionally, we investigated the effect of MS therapy on the presence of Abs targeting LAM as the major mycobacterial antigen purified from the MAP 1515 strain.

The presence of Abs directed against the selected peptides was assessed through an optimized protocol for indirect enzyme-linked immunosorbent assay (ELISA) employed as previously described [18,20] using 10 ng/μL peptide concentration for plate coating. Optical density values read at a wavelength of 405 nm were normalized to a highly responsive serum included in each experiment with the maximum reactivity established at 1.0 arbitrary units (AU)/mL. The mean of two technical replicates was considered for further data analysis.

### 2.3. Statistical Analysis

Statistical analysis was performed using GraphPad Prism 8.0 software (GraphPad Software Inc., La Jolla, CA, USA). After determining sample distribution through the D’Agostino–Pearson normality test, the comparison between MS and control groups was executed using a two-tailed Mann–Whitney *U* test with *p* < 0.05 considered statistically significant. The positivity threshold was set at 0.51 (AU)/mL for IL-2_56–70_, 0.62 (AU)/mL for IL-2_6–20KK_, 0.64 (AU/mL) for HERV-Wenv-su_93–108_, and 0.46 (AU/mL) for HERV-Wenv-su_248–262_ based on the receiver operating characteristic (ROC) curve with ≥ 90% specificity and 95% confidence interval. The percentage of positive subjects in both groups was assessed through Fisher’s exact test. Patients classified in age-related groups were compared using ANOVA. Correlations between levels of peptide-specific Abs and clinical or demographic variables were determined by principal component analysis (PCA) using XLSTAT software ver. 17 (Addinsoft, New York, NY, USA).

## 3. Results

**Figure 1:**
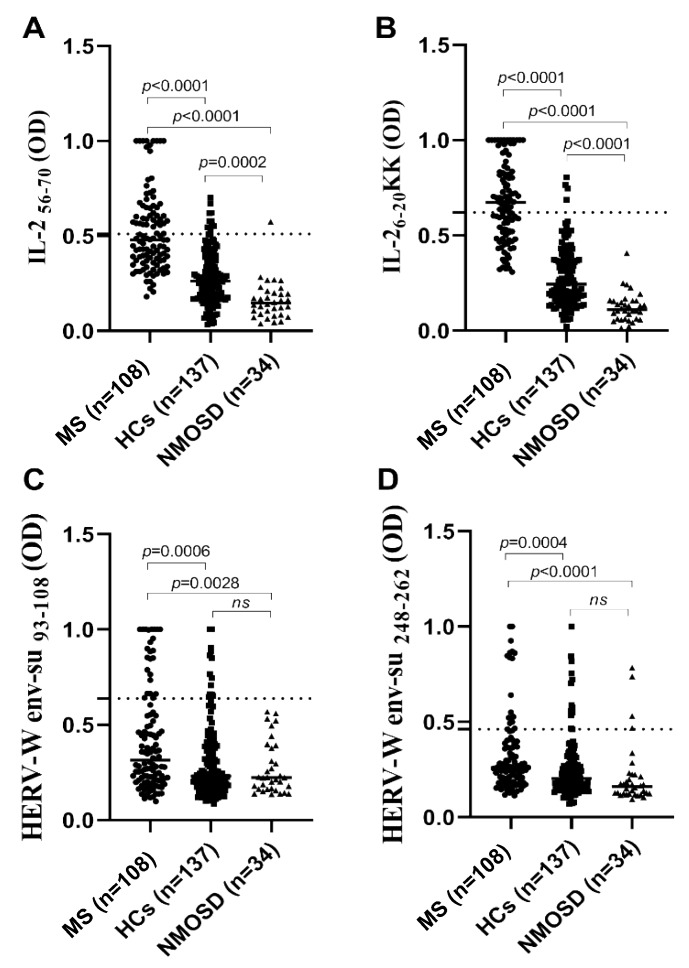
(**A–D**) Abs reactivity of MS, NMOSD, and healthy subjects against peptides derived from interleukin 2 (IL-2) (**A**,**B**) and human endogenous retrovirus type W (HERV-W) (**C**,**D**). Values relative to statistical differences between groups are reported above each distribution. Dotted lines correspond to the positivity threshold established for each peptide based on the receiver operating characteristic (ROC) curve analysis.

Seroreactivity elicited by IL-2_6–20KK_ was higher in the MS group compared to healthy subjects reaching 58.33% of patients whose autoreactive Abs exceeded positivity threshold, while elevated responses against IL-2_56–70_ were registered in 43.52% of MS individuals (Figure 1). Abs prevalence among control subjects was significantly lower for both peptides with respective positivity observed in 2.92% and 8.03% of samples. For each peptide, the comparison between MS and controls by analyzing either mean seroreactivity values or the number of positive patients was highly significant (*p* < 0.0001). When considering anti-IL-2 Abs overlap, 37.04% of MS and two control subjects showed double positivity that was reflected by correlation coefficients (*R*^2^ = 0.4958 and *R*^2^ = 0.1926, respectively). In contrast, NMOSD patients showed significantly lower Abs responses to IL-2 peptides compared to both MS and healthy controls, with only one sample positive to IL-2_56–70_ (Figure 1).

Overall trends observed for anti-IL-2 reactivity were maintained for HERV-W-derived epitopes; however, the difference regarding the percentage of positive samples between groups was less pronounced (Figure 1). Among MS patients, 21.3% were positive to HERV-Wenv-su_93–108_ and 15.74% to HERV-Wenv-su_248–262_, and in both tests the difference was significant when comparing to healthy controls (*p* = 0.0006 and *p* = 0.0004, respectively), who reached 9.49% of positive subjects for each peptide. Although NMOSD reactivity was lower with respect to the other groups with no samples exceeding the positivity threshold for HERV-Wenv-su_93–108_ and 11.76% of samples positive to HERV-Wenv-su_248–262_, statistical significance was attained only upon comparison with results relative to MS (*p* = 0.0028 and *p* < 0.0001, respectively; Figure 1).

**Figure 2:**
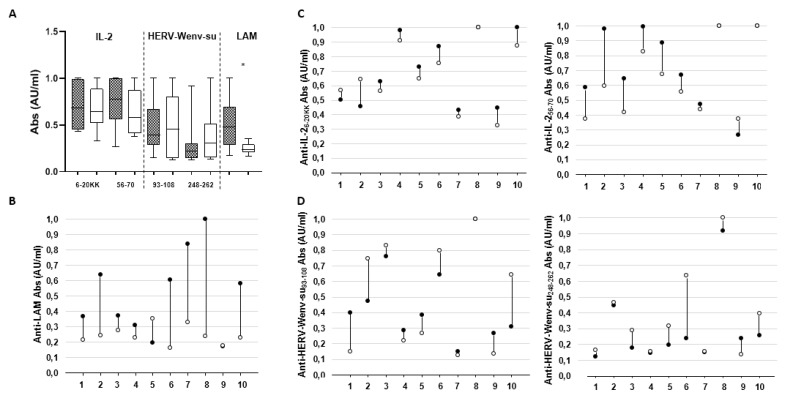
Changes in antibody (Abs) profiles of MS patients after a one-year follow-up. Overall Abs levels specific for each antigen are shown along with corresponding peptide position indicated at the base (**A**). Changes in Abs response to lipoarabinomannan (LAM) glycolipid (**B**), as well as IL-2 (**C**) and HERV-Wenv-su (**D**) peptides are also shown for single patients. Dark grey bars and black dots correspond to T0 sampling, while light grey bars and white dots indicate values after 12 months of MS therapy or the lack of therapy. On the *X*-axis, patient reference numbers are reported.

Upon evaluation of Abs status in follow-up samples, a slight but not significant decrease of seroreactivity to both IL-2 peptides was observed after a one-year therapy (Figure 2). Contrarily, therapy seemed not to have had significant overall effects on the levels of Abs targeting HERV-W epitopes, which increased only slightly, and the large span of Abs levels was mainly due to untreated patients. In light of findings that some immuno-modulating drugs administered to alleviate symptoms of chronic inflammatory disorders, such Crohn’s disease, may exert inhibiting action on mycobacteria [23,24], we evaluated serological responses against MAP-purified LAM, observing a striking decrease of relative Abs (*p* = 0.018).

**Figure 3:**
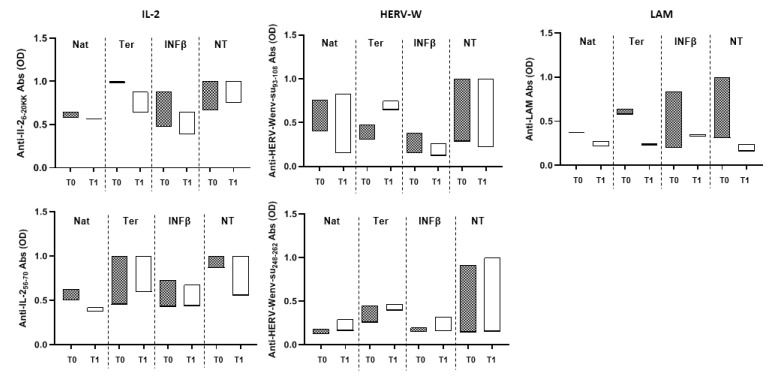
The effect of MS therapy on Abs against IL-2, HERV-W, and LAM peptides. The presence of Abs was assessed at sample collection (T0) and after one year (T1) according to administered MS therapy. Nat: natalizumab (*n* = 2); Ter: teriflunomide (*n* = 2); INFβ: interferon beta (*n* = 2); NT: no therapy (*n* = 3). The analysis is based only on therapies for which follow-up samples of at least two patients were available.

We further investigated trends in association with the type of administered treatment (Figure 3), which was unvaried for natalizumab and teriflunomide or slightly decreased for INF-β when evaluating anti-IL-2_6–20KK_ Abs. Responses against IL-2_56–70_ were decreased independently of the type of therapy, even though they corresponded to highly elevated Abs levels in patients treated with teriflunomide. The lack of therapy was associated with elevated responsiveness to both IL-2 epitopes. Regarding HERV-W peptides, all therapies or the lack thereof elicited slight variations in Abs levels with trends following increasing patterns rather than a reduction in seroreactivity. Only anti-LAM Abs were strongly reduced by each type of therapy but also when no treatment was administered (Figure 3).

## 4. Discussion

The presence of anti-IL-2 Abs may lead to a drastic reduction of IL-2 concentrations, thus their role in disrupting the balance of immune responses may be decisive for the onset of immune-mediated diseases. The effects of anti-IL-2 Abs must be investigated in this context. If IL-2 activity decreases to extremely low levels due to high titers of anti-IL-2 Abs, it leads to inhibition of activated immune cells, increase of regulatory natural killer cells, effects on dendritic cells, and inhibition of innate lymphoid tissue inducer cells. In the opposite case, if anti-IL-2 Abs are ineffective, we may observe an increase of the myelin-reactive T-cell population characterized by their ability to produce large amounts of IL-2 [25]. Of interest, daclizumab, a monoclonal antibody specific for the IL-2R α-chain (CD25), has been used to treat and modulate immune responses in relapsing-remitting MS [26]. However, episodes of meningoencephalitis and drug reaction with eosinophilia and systemic symptoms (DRESS) reported after daclizumab therapy have led to its further suspension in March 2018 [27].

Our study demonstrated that, in the tested group, MS patients were more likely to display elevated levels of anti-IL-2 Abs compared to healthy subjects. This difference was highly significant with respect to NMOSD patients who developed low mean values corresponding to the assessed Abs in general. In the initial disease phases, NMOSD presents similar symptoms to MS with the difference that the former involves immune-mediated demyelination and axonal damage targeting the AQP4 water channel predominantly contained within optic nerves and spinal cord, whereas the latter affects also the brain. As not all NMOSD patients develop anti-AQP4 Abs and false-positive results may be obtained in the case of other inflammatory diseases of the central nervous system (CNS) [28], the diseases are often mistakenly diagnosed and improperly treated, resulting in a rapid accumulation of disability. Therefore, patterns involving Abs against IL-2 and HERV-Wenv may be useful for an additional screening to confirm diagnostic outcomes based on standard criteria.

The effect of a 12-month therapy visible for most patients showed discreet but opposite trends for IL-2 and HERV-Wenv-su peptides. Responses to distinct therapeutic approaches evaluated here were specific to single antigens rather than to disease-modifying agents. This may be related to differences in molecular nature and physiological role between IL-2 and HERV-W. Especially for IL-2, immunomodulatory drugs are expected to induce stronger responses by targeting their action on components of the immune system. A marked decrease of seroreactivity towards LAM in patients who did not follow any therapy may occur as a spontaneous regression similar to natural healing observed in pulmonary tuberculosis [12] and linked to the return of MAP to latent infection phases in concert with MS relapse recovery. Even though the course of MAP infection in humans is unclear, typical symptoms of paratuberculosis affecting ruminant animals consist of transient active disease associated with a massive production of cytokines including IL-2 and intervals when the mycobacterium assumes intracellular phenotype [29]. Any link between MAP and HERV-W involving retroviral transactivation in MS remains to be established.

In light of our previous findings detecting a significant reduction of HERV-Wenv expression following a 24-month natalizumab treatment [18], it is plausible that a 12-month long therapy may be insufficient to induce a similar drop of relative Abs or that therapeutic approaches are less efficient in the assessed study population. This issue requires further investigation. The impact of HERV-W and MAP on MS relapse will be the object of future assessment including retroviral expression and detection of other mycobacterial antigens or markers with regard on IL-2 levels and related Abs.

The small number of follow-up samples is a limitation of this study, although the genetic homogeneity and the availability of post-therapy samples are of great advantage. Even though data based on populations originating from Sardinian areas characterized by high MS prevalence and by a probable intrinsic risk supposedly due to long-lasting genetic isolation [30] provide a genetically homogeneous study group, probably exposed to a common environmental factor, respective Abs levels should be evaluated in cohorts with different biogeographical background. This will shed light on the strength with which genetic factors may influence the development of MS and increase the possibility to predict the disease for certain genotypes.

## Figures and Tables

**Table 1:** Demographic and clinical characteristics of multiple sclerosis (MS) patients, neuromyelitis optica spectrum disorder (NMOSD) patients, and healthy controls (HCs).

Clinical Data	MS *n* = 108	NMOSD *n* = 34	HCs *n* = 137
Age, years	40.06	51.32	46.30
Female, *n*	66	29	90
Male, *n*	42	5	47
AQP4-Abs −		11	
AQP4-Abs +		23	
Cortisone	25		
No cortisone	18		
Interferon beta	6		
Alemtuzumab	3		
Dimethylfumarate	5		
Teriflunomide	5		
Ocrelizumab	1		
Natalizumab	2		
Fingolimod	4		
No therapy	39		
EDSS	2.53 ± 2		
RRMS, *n* (%)	34 (31.48)		
SPMS, *n* (%) Onset, *n* (%)	4 (3.7) 70 (64.81)		

MS: multiple sclerosis; NMOSD: neuromyelitis optica spectrum disorder; HCs: healthy controls; AQP4-Abs: anti-aquaporin-4 antibodies; EDSS: Expanded Disability Status Scale; RRMM: relapsing-remitting MS; SPMS: secondary progressive MS.
